# Increased frontal gray matter volume in individuals with prodromal psychosis

**DOI:** 10.1111/cns.13143

**Published:** 2019-05-25

**Authors:** Xiao‐Xiao Shan, Yang‐Pan Ou, Pan Pan, Yu‐Dan Ding, Jin Zhao, Feng Liu, Jin‐Dong Chen, Wen‐Bin Guo, Jing‐Ping Zhao

**Affiliations:** ^1^ Department of Psychiatry The Second Xiangya Hospital of Central South University Changsha China; ^2^ National Clinical Research Center on Mental Disorders Changsha China; ^3^ Department of Radiology Tianjin Medical University General Hospital Tianjin China

**Keywords:** cognitive function, gray matter volume, magnetic resonance imaging, prodromal psychosis, support vector machine

## Abstract

**Background:**

Brain anatomical deficits associated with cognitive dysfunction have been reported in patients with schizophrenia. However, it remains unknown whether such anatomical deficits exist in individuals with prodromal psychosis. The present study is designed to investigate anatomical deficits in prodromal individuals and their associations with clinical/cognitive features.

**Methods:**

Seventy‐four prodromal individuals and seventy‐six healthy controls were scanned using structural magnetic resonance imaging. Support vector machines were applied to test whether anatomical deficits might be used to discriminate prodromal individuals from healthy controls.

**Results:**

Prodromal individuals showed significantly increased gray matter volume (GMV) in the right inferior frontal gyrus (IFG) and right rectus gyrus relative to healthy controls. No correlations were observed between increased GMV and clinical/cognitive characteristics. The combination of increased GMV in the right rectus gyrus and right IFG showed a sensitivity of 74.32%, a specificity of 67.11%, and an accuracy of 70.67% in differentiating prodromal individuals from healthy controls.

**Conclusion:**

Our results provide evidence of increased frontal GMV in prodromal individuals. A combination of GMV values in the two frontal brain areas may serve as potential markers to discriminate prodromal individuals from healthy controls. The results thus highlight the importance of the frontal regions in the pathophysiology of psychosis.

## 1 INTRODUCTION

Individuals with prodromal psychosis (also known as the “ultra‐high‐risk” subjects), along with various cognitive deficits, such as executive functions, processing speed, working memory, and attention,^1^, ^2^ are known to be on the pre‐onset stage of psychosis and thus show potentially prodromal psychotic symptoms, which may (or may not) progress to full‐blown psychosis.^3^ A meta‐analysis showed that prodromal individuals had a mean (95% CI) risk of transiting to psychosis of 29% (23%‐36%) over the period of 2 years of follow‐up, 32% (24%‐35%) over the period of 3 years, and 36% (30%‐43%) after 3 years.^4^ However, a recent study showed a low psychosis incidence in the prodromal group after 12 months of follow‐up, and the low incidence might be due to a short follow‐up time.^5^ The pathophysiological mechanism of prodromal psychosis still requires further exploration because of the inconsistencies in these field advancements.

Some studies observed that prodromal individuals had reduced GMV in several brain regions, particularly in the hippocampal gyrus,^6^ lateral temporal lobe,^7^ and prefrontal cortex(PFC),^7^, ^8^ including the medial PFC and lateral PFC. Previous review revealed that prodromal individuals exhibited reduced GMV in the temporal gyrus, PFC, and anterior cingulate cortex (ACC) before illness onset.^9^ Nenadic et al found reduced GMV in the right middle/superior temporal, left superior frontal, and right postcentral cortices in prodromal individuals compared with healthy controls. Meanwhile, they also found increased GMV in the left temporal gyrus in prodromal individuals.^10^ However, several neuroimaging studies found no GMV reduction between prodromal individuals and controls.^11^, ^12^ A recent study found no significant structural changes in the prodromal individuals, but patients with first‐episode schizophrenia exhibited significantly decreased GMV in the bilateral superior parietal lobule and left orbital frontal cortex compared with prodromal individuals and healthy controls.^13^ Hence, it remains controversial whether prodromal individuals have structural alterations.

Several factors may attribute to the inconsistent findings. First, sample size, clinical characteristics, and analysis methods differ across studies. For example, a recent study indicated that a voxel‐based morphometry (VBM) analysis by Computational Anatomy Toolbox (CAT12) was more accurate and robust against volumetric alterations compared with the VBM8 toolbox.^14^ Furthermore, a small sample size may confine the power to detect volumetric differences. Second, medication use can confound the results across studies. For example, previous studies have reported that antipsychotic treatment might decrease GMV in the temporal and frontal areas in early phases of psychosis.^15^ Therefore, it is meaningful to conduct a structural study to examine whether prodromal individuals have GMV deficits after controlling for the abovementioned confounding factors.

The prediction of psychosis based on neuroanatomical biomarkers is possible by using multivariate pattern recognition approaches, including support vector machine (SVM). SVM has emerged as a promising tool for diagnostic purpose of various neuropsychiatric conditions.^16^ Previous SVM results showed that the classification pattern included the prefrontal and temporal cortices, as well as a large bilateral cluster containing the parahippocampus and hippocampus where GMV reductions were recognized in the prodromal individuals.^17^, ^18^ SVM could successfully discriminate prodromal individuals from healthy controls with an accuracy of 68.42% based on structural magnetic resonance imaging (MRI) and diffusion tensor neuroimaging parameters.^19^ Zarogianni et al^20^ reported an accuracy of 74% for predicting later onset of psychosis, and the discriminative neuroanatomical pattern included many brain areas such as the temporal, frontal, and parietal regions. Therefore, SVM may be feasible in the early discrimination of psychosis using the neuroanatomical‐based pattern recognition method.

In the present study, a relatively large sample of prodromal individuals was recruited. Prodromal individuals were drug‐naive to eliminate the effects of medication use. Structural data were analyzed with the CAT12 method with optimized segmentation and normalization. Based on the abovementioned studies, we hypothesized that prodromal individuals would exhibit significantly decreased GMV in certain brain regions, especially in the prefrontal and temporal regions, which could be applied as potential image markers to identify prodromal individuals from healthy controls using SVM. We also hypothesized that decreased GMV would be significantly correlated with clinical/cognitive features.

## 2 MATERIALS AND METHODS

### 2.1 Participants

Seventy‐four prodromal individuals from the Department of Psychiatry, the Second Xiangya Hospital of Central South University in China were enrolled in the study. All prodromal individuals were recruited from the outpatient department. There are eight prodromal individuals in the brief intermittent psychotic syndrome (BPS) subcategories, forty‐four prodromal individuals in the attenuated positive symptom syndrome (APS) subcategories, eleven prodromal individuals in the genetic risk and deterioration syndrome (GRD) subcategories, and eleven prodromal individuals met the criteria of two prodromal syndromes (APS and GRD), respectively. Seventy‐six healthy controls unrelated to prodromal individuals were recruited from the local community. Healthy controls and their first‐degree relatives had no history of psychiatric disorders. All subjects were right‐handed, aged 13‐39 years, and had at least a junior high school education level with the ability to understand the survey contents. Age, sex, and years of education were matched between prodromal individuals and healthy controls. The prodromal individuals were screened using the structured interview for prodromal syndromes (SIPS) and scale of prodromal syndromes (SOPS),^21^ including (a) BPS, (b) APS, and (c) GRD. The SIPS (19 items) contains four symptom clusters: negative symptoms; positive symptoms; disorganized symptoms; and general symptoms. The SOPS was used to identify the presence of a psychotic syndrome that was either (a) disorganizing or dangerous or (b) existing at least an hour within a day on average 4 days of a week for at least 1 month. The reliability and validity of the SIPS/SOPS were acceptable.^21^, ^22^ Healthy controls were screened by the non‐patient version of the Structured Clinical Interview for DSM‐IV. All individuals were drug‐naive.

Cognitive function was evaluated using the Brief Assessment of Cognition in Schizophrenia Symbol Coding Test (BACS‐SC), Hopkins Verbal Learning Test‐Revised (HVLT‐R), Brief Visuospatial Memory Test‐Revised (BVMT‐R), Stroop Color Word Test (SCWT), Trail Making Test A (TMT‐A), and Continuous Performance Test (CPT). BACS‐SC is used to measure processing speed and attention.^23^ HVLT‐R is a list of learning verbal memory test including 12 words to assess verbal memory.^24^ BVMT‐R is widely utilized to evaluate visuospatial learning and memory in neuropsychological assessment.^25^SCWT is applied to evaluate the attention and working memory functions, which includes three parts. Part 1 is about reading a list of 100 words, and the words “red,” “green,” or “blue” are printed in black. Part 2 requires the participants to distinguish the ink color of a list of unmeaning characters. Part 3 requires the participants to report the ink color of the words “red,” “green,” and “blue.”^26^TMT‐A is applied to measure psychomotor speed, including 25 circles (numbered 1‐25) distributed over a piece of paper. Participants are required to draw lines to link the numbers as quickly as possible in an ascending order.^27^ CPT is a widely used measure of sustained attention.^28^ These tests cover visual learning and memory, verbal processing speed, attention/vigilance, and executive function.

Exclusion criteria for all participants were any physical illnesses, such as liver and kidney diseases, cardiovascular diseases, and any past or present neuropsychiatric disorders; any traumatic brain injury; seizures; drug or alcohol abuse or dependence; pregnancy; and any contraindications to MRI scan.

The Ethics Committee of the Second Xiangya Hospital of Central South University approved this study. After a complete explanation, all participants (if the subject was under 18 years of age, the signature of the guardian was required) submitted their written informed consent.

### 2.2 Scan acquisition

Magnetic resonance imaging scanning was conducted with a 3.0 T Siemens scanner (General Electric). The participants were told to lie supine and stay still with eyes closed. Foam pads and soft earplugs were used to reduce scanner head motion and noise. A 3D magnetization‐prepared rapid acquisition gradient‐echo sequence was used with the following parameters: repetition time of 2710 ms, echo time of 3.78 ms, flip angle of 7°, inversion time of 1000 ms, slice thickness of 1 mm, field of view of 256 mm × 256 mm, matrix of 256 × 256, no gap, and 188 slices.

The structural MRI data were preprocessed using the CAT12 (http://dbm.neuro.unijena.de/cat) method of the Statistical Parametric Mapping software package (SPM12, http://www.fil.ion.ucl.ac.uk/spm/software/spm12/). The data analysis was conducted as follows. (a) All images were reoriented to the same origin point and spatial orientation, and the non‐linear deformation field was reckoned that prime overlaid the probability maps of tissue on the individual images. Three tissue components, including cerebral spinal fluid, gray matter, and white matter, were classified. (b) Registering the native‐space tissue segments to the standard Montreal Neurological Institute template by the affine registration algorithm and correcting the differences in the individuals’ head positions or orientation during MRI scanning. (c) The diffeomorphic anatomical registration was used to all individuals’ gray matter to refine the inter‐individual registration by the exponentiated lie algebra (DARTEL) toolbox. (d) Modulating the intensity of the gray matter images with the surrounding voxels compressed or expanded. Comparing the relative GMV corrected for individual brain size, gray matter tissues were modulated by a non‐linear deformation approach. (e) When the preprocessing pipeline was completed, the quality check using a CAT12 toolbox was performed to evaluate the homogeneity for the gray matter tissues. (f) The gray matter tissue segments were smoothed with an 8 mm Gaussian Kernel for the group analysis. This step contributed to increase the signal‐to‐noise ratio and decrease the influence of misregistration between images and benefit on the statistical normality. (g) Using the automated anatomical labeling atlas software and anatomical atlases to determine the most significant clusters.

### 2.3 Statistical analysis

The clinical and demographic data of the two groups were compared by two‐sample *t* tests or a chi‐square test when necessary.

The differences of GMV between prodromal individuals and healthy controls were compared using voxel‐wise two‐sample *t* tests, with total intracranial volume, age, and years of education as covariates of no interest. The significance level was set at *P* < 0.05 corrected according to the Gaussian random field theory (voxel significance: *P* < 0.001, cluster significance: *P* < 0.05) for multiple comparisons with the REST software.

Once significant differences in GMV were observed in brain regions between the two groups, the mean GMV values were extracted from those brain regions. Pearson's correlation analyses between abnormal GMV and clinical/cognitive parameters were carried out with threshold of *P* < 0.05. Bonferroni correction was used to limit type I error.

### 2.4 Classification analysis

To test the capacity of the combination of abnormal GMV in any two brain regions to discriminate the prodromal individuals from the controls, we applied a SVM ran in MATLAB using the LIBSVM software package (http://www.csie.ntu.edu.tw/~cjlin/libsvm/). The “leave‐one‐out” method was employed in the study.

## 3 RESULTS

### 3.1 Demographic and clinical characteristics

Table 1 presents details of the demographic and clinical characteristics. No significant differences are noted in age and sex ratios between prodromal individuals and healthy controls. Significant differences are observed in years of education (11.82 ± 2.94 vs. 15.11 ± 1.8, *P* < 0.001). The scores of the BACS‐SC, HVLT‐R, CPT, BVMT‐R, and SCWT are significantly lower in prodromal individuals than those in healthy controls. Prodromal individuals score significantly higher than healthy controls in the TMT‐A scores.

**Table 1:** Characteristics for prodromal individuals and healthy controls

	Prodromal individuals (n = 74)	Controls (n = 76)	*P* value
Sex (male/female)	43/31	39/37	0.403^a^
Age (y)	22.0 ± 5.25	21.6 ± 2.97	0.523^b^
Years of education (y)	11.8 ± 2.94	15.1 ± 1.88	<0.001^b^
SIPS			
Positive symptoms	9.51 ± 4.53		
Negative symptoms	11.4 ± 6.03		
Disorganized symptoms	5.05 ± 2.93		
General symptoms	5.72 ± 3.17		
Total scores	32.0 ± 11.1		
TMT‐A	39.5 ± 17.1	34.0 ± 10.3	0.022^b^
BACS: symbol coding	54.4 ± 10.2	61.8 ± 9.34	<0.001^b^
HVLT‐R	24.2 ± 5.84	26.9 ± 3.96	0.002^b^
BVMT‐R	23.0 ± 10.0	27.6 ± 6.95	0.002^b^
Stroop Word	86.6 ± 27.2	98.1 ± 17.3	0.014^b^
Stroop Color	56.5 ± 19.2	72.7 ± 13.6	<0.001^b^
Stroop Color and Word	33.2 ± 13.4	43.5 ± 8.38	<0.001^b^
CPT	2.40 ± 0.79	2.85 ± 0.56	<0.001^b^

Abbreviations: BACS, Brief Assessment of Cognition in Schizophrenia; BVMT‐R, Brief Visuospatial Memory Test‐Revised; CPT, Continuous Performance Test; HVLT‐R, Hopkins Verbal Learning Test‐Revised; SIPS, structured interview for prodromal syndromes; TMT‐A, Trail Making Test, part A.

a The *P* values for sex distribution were obtained by a chi‐square test.

b The *P* values were obtained by two‐sample *t* tests.

### 3.2 Differences in GMV between prodromal individuals and healthy controls

**Figure 1:**
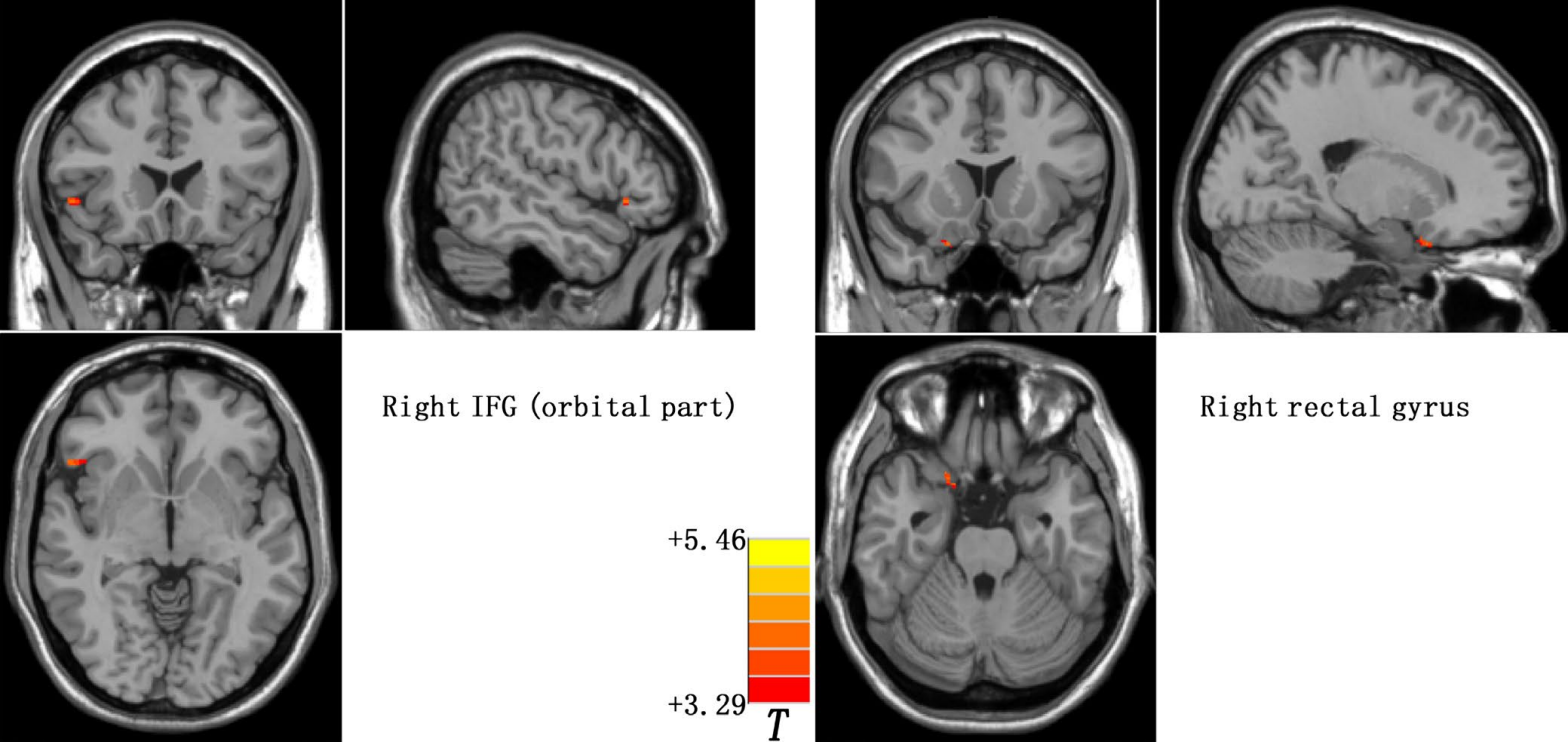
GMV differences between prodromal individuals and healthy controls. Increased GMV in the right IFG and right rectal gyrus were observed in the prodromal individuals. The color bar represents the *t* values of the group analysis of GMV. GMV, gray matter volume; IFG, inferior frontal gyrus

Relative to healthy controls, prodromal individuals exhibit significantly increased GMV in the right inferior frontal gyrus (IFG) (*t* = 4.1821) and right rectus gyrus (*t* = 4.0674). Table 2 and Figure 1 present the detailed information.

**Table 2:** Increased GMV in prodromal individuals relative to controls

Cluster location	Peak coordinate			Cluster size (voxel)	*t* value
	*x*	*Y*	*z*		
Right IFG (orbital part)	52.5	21	−4.5	26	4.1821
Right rectal gyrus	16.5	12	−25.5	30	4.0674

Abbreviations: GMV, gray matter volume; IFG, inferior frontal gyrus.

The significance level was set at *P* < 0.05 corrected by the Gaussian random field (GRF) theory (voxel significance: *P* < 0.001, cluster significance: *P* < 0.05) for multiple comparisons with the REST software.

### 3.3 Correlation analysis

No significant correlations are observed between increased GMV and clinical characteristics/cognitive function in prodromal individuals (*P* > 0.05, Bonferroni corrected).

### 3.4 Distinguishing prodromal individuals from healthy controls

**Figure 2:**
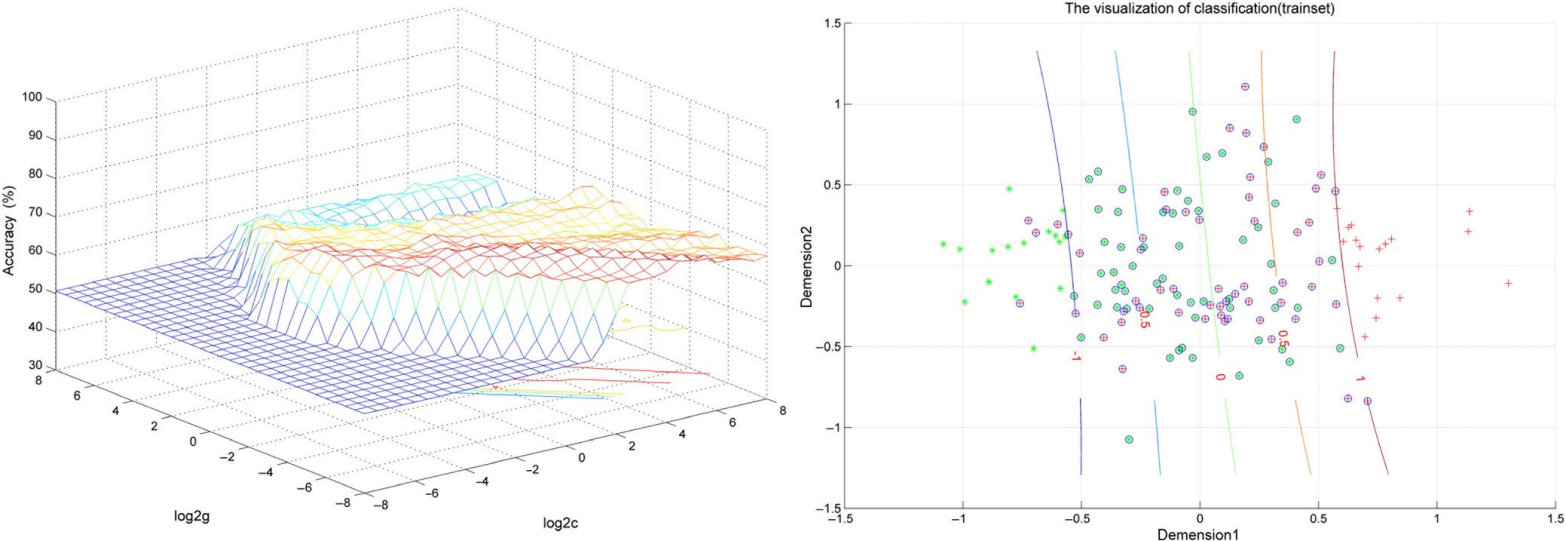
Visualization of classification by the method of support vector machine (SVM) using the combination of the GMV values in the abnormal brain regions. In the right of figure, dimension 1 and 2 represent the GMV values in the right IFG and right rectal gyrus, respectively. Red crosses represent the controls, and green crosses represent the prodromal individuals. GMV, gray matter volume; IFG, inferior frontal gyrus

The combination of the GMV values in the right rectus gyrus and right IFG showed a sensitivity of 74.32% (55 of 74 in the prodromal group), a specificity of 67.11% (51 of 76 in the control group), and an accuracy of 70.67% (106 of 150 in the two groups), as shown in Figure 2.

## 4 DISCUSSION

By analyzing the whole brain GMV with CAT12, prodromal individuals showed significantly increased GMV in the right IFG and right rectus gyrus compared with healthy controls. No correlations were noted between increased GMV in the two brain regions and clinical characteristics/cognitive function. Moreover, the SVM analysis showed that a combination of the GMV values in these two brain regions might be a potential marker to distinguish prodromal individuals from controls.

Our findings of increased GMV in the right IFG and right rectus gyrus in prodromal individuals are inconsistent with our hypothesis and the results of most previous high‐risk studies, which showed decreased volume in the frontotemporal regions in the prodromal individuals.^10^, ^29^ The present results are also inconsistent with the results of previous Asian high‐risk studies. Some VBM studies from Asian reported GMV reductions in the frontotemporal regions in the prodromal individuals compared with healthy controls.^7^, ^30^ Other studies from Asian even failed to find significant difference in the regional GMV between the prodromal individuals and healthy controls.^13^, ^31^ Several factors deserve consideration in explaining the regionally increased GMV seen in the present study. First, age range of prodromal individuals may be considered. Previous studies reported different maturational trajectories in different brain regions, which presented an inverted U‐curve with maximal point in adolescents.^32^ Prodromal individuals in the present study were at the stage of adolescents. Brain gray matter maturation might be halted at the maximal point of the inverted U‐curve in prodromal individuals, which might result in increased frontal gray matter in prodromal individuals in the present study. Second, prodromal individuals are at the very early stage of psychosis. Very early‐stage neuronal pathology, such as hypertrophy or preapoptotic osmotic changes, could possibly increase regional volumes.^33^ The expectation is supported by a longitudinal study with increased GMV in the right ACC, IFG, and left cerebellum in subjects at clinical risk for psychosis.^34^ Third, medication use might confound previous studies.^12^, ^35^ Progressive GMV loss was reported after antipsychotic treatment.^36^, ^37^ Also, antipsychotic medications could contribute to the decline of brain tissue volumes in animal studies.^38^, ^39^ Vernon et al^38^ reported that chronic exposure (8 weeks) to antipsychotic drugs in rats would induce significant decreases in the whole‐brain volume, mainly in the frontal cortex volume. which could be normalized after withdrawal of the antipsychotic medications.^40^ Prodromal individuals in the present study were drug‐naive, and thus confounding effects induced by antipsychotic medications could be limited. Therefore, it is expected that prodromal individuals in this study presented increased GMV. Fourth, sample size is relatively large in the present study, which may have statistical power to identify hypertrophic effects not indicated frequently in previous studies. Fifth, CAT12 is an advanced method with optimized segmentation and normalization, which is more sensitive in the analysis of GMV than the previous version such as the VBM8 method. Other potential reasons are neuroplasticity. According to results from postmortem studies, alterations in GMV may be related to changes in dendritic density, synaptic, and neuronal, as well as increased afferentation in certain regions.^41^ Sowell et al^42^ suggested that different cortical GMV might be due to the differences in the neuropil, where synaptic connections were formed between axons and dendrites. Therefore, different synaptic pruning during adolescence might cause the different structural changes between prodromal individuals and controls. Finally, some studies reported that the inflammatory and immune mechanisms might be related to brain structures, which directly influenced neuronal proliferation, migration, differentiation, and apoptosis. Whitford et al^43^ found that prodromal individuals exposing herpes simplex virus type 1 (HSV1) had GMV abnormalities in the cuneus, which was in line with the region found in established schizophrenic patients with HSV1‐infected. By contrast, some studies have also showed increased GMV in prodromal individuals, including a report of increased GMV in the left parietal/posterior temporal region in prodromal individuals.^44^ Fusar‐Poli et al^34^ also found that increased GMV in the right ACC and IFG in prodromal individuals. In line with those studies, our findings provide important information of increased frontal GMV in the pathophysiology of psychosis.

The frontal cortex has well‐established effects on the processing of cognitive and mnemonic activities.^45^ The encoding‐related activity of the medial‐temporal lobe and the interaction with the inhibition‐correlation activity of the right frontal cortex mediate intentional forgetfulness.^46^ A recent review indicated that changes in cortical midline structures during prodrome may be correlated to a disrupted sense of self, on the basis of the involvement of the ACC and medial PFC regions in self‐related processing.^47^ Our previous study found that prodromal individuals had altered functional connectivity strength (FCS) in the frontal‐occipital network. Furthermore, decreased FCS in the left middle frontal gyrus was significantly correlated with the cognitive measures in the prodromal individuals.^48^ Hence, correlations between increased GMV in the frontal lobes and clinical/cognitive parameters are expected in the present study. However, no correlations were somewhat surprised in the present study. Increased GMV in the frontal lobes might be trait alterations independently of symptom severity and cognitive deficits in the prodromal individuals.

A few longitudinal studies revealed that prodromal subjects showed progressive GMV decreases over time. Prodromal subjects, who later developed psychosis, showed active GMV loss in several brain regions, including the prefrontal cortices, superior temporal gyrus, and parahippocampal gyrus during the transition period.^29^, ^49^ Recent multicenter study using a relatively large prodromal subjects cohort who developed psychosis, also showed progressive GMV loss mainly in the prefrontal regions, superior temporal, parietal, and parahippocampal regions.^50^ Taken together, the current evidence revealed that the progressive pathological process precedes the first manifestation of overt psychosis in the prefrontal and other brain regions.

Previous study showed that more than 0.7 of specificity or sensitivity is good for establishing diagnostic index,^51^ whereas less than 0.6 of specificity or sensitivity may be poor for diagnostic indicator.^52^ SVM has been well applied in extensive biomedical applications for diagnostic purpose.^53^, ^54^ Koutsouleris et al^55^ showed an accuracy of 86% for distinguishing prodromal individuals from controls using the SVM analysis. Then, they found the classification accuracy between healthy controls and prodromal individuals without a subsequent disease conversion was 66.9% in another independent population.^56^ A recent MRI study found that structural MRI data allowed identification of prodromal individuals with a specificity of 76%, a sensitivity of 68%, and an accuracy of 72% with the SVM analysis.^17^ The present SVM analysis exhibited that the combination of GMV values in the right IFG and right rectus gyrus showed an accuracy of 70.67% in discriminating prodromal individuals from healthy controls. Hence, the combination of an increased GMV values in the right IFG and right rectus gyrus could serve as a potential image marker to distinguish prodromal individuals from controls.

Cognitive deficits may precede the onset of psychosis, which may be helpful as potential markers of increased vulnerability for psychosis. The present study compared neurocognitive performance between prodromal individuals and healthy controls. We found that the scores of the BACS‐SC, HVLT‐R, CPT, BVMT‐R, and SCWT were significantly lower in prodromal individuals than those in healthy controls, suggesting that prodromal individuals had cognitive deficits, such as decreased processing speed, attention, executive function, visual learning, and memory. The results were consistent with previous findings, which revealed that prodromal psychosis status was related to impairments in multiple neurocognitive components, including learning, attention, memory, executive function, and processing speed.^57^, ^58^ A recent study showed that cognitive deficits in prodromal individuals were intermediate between FES and first‐degree relatives of psychosis groups.^2^ Moreover, Ucok et al^59^ found that the cognitive performance of prodromal individuals was similar to that of FES. Previous meta‐analysis also suggested that prodromal individuals were significantly impaired in various cognitive function domains.^60^ Together with the abovementioned studies, the present study showed cognitive deficits in prodromal individuals.

Several limitations exist in the present study. First, the study is cross‐sectional. It is unclear how many of the prodromal individuals will transition to psychosis in the follow‐up period, and whether increased GMV in the frontal lobes will be stable or decrease over time. Future longitudinal research is necessary to examine this possibility. Second, this study did not recruit patients with schizophrenia to compare their anatomical changes with those of prodromal individuals. This issue might limit our comprehending of the disease progression between prodromal individuals and patients with schizophrenia. Finally, level of education is unmatched between two groups. Cognitive differences may be driven by psychotic pathology of unmatched level of education. Although we tried to minimize the possible effects of unmatched level of education using it as a covariate of no interest in the analyses, the effects of unmatched level of education might not be completely eliminated. Therefore, the samples differ in terms of level of education and cognitive test performance which may have confounded the results.

In conclusion, the present study provides evidence of increased GMV in the frontal gyri in prodromal individuals. A combination of GMV values in these two brain areas may serve as potential markers to discriminate prodromal individuals from healthy controls. The results thus highlight the importance of the frontal regions in the pathophysiology of psychosis.

## CONFLICT OF INTEREST

We declare that none of the authors holds any actual or potential conflict of interest for this study.
